# Weekly Physiological Changes in Blood Pressure During Three Weeks Daily Consumption of 10 Grams of Cocoa Powder Among Young Black Africans in Côte d’Ivoire

**DOI:** 10.3389/fphys.2021.634791

**Published:** 2021-02-18

**Authors:** Edwige Siransy-Balayssac, Soualiho Ouattara, Hugues Ahiboh, Toh Bi Youzan, Fagnan Levy Gouh, Koffi Bertrand Yao, Mocket Ehouman, Cyrille Serges Dah, Pascal Bogui

**Affiliations:** ^1^Laboratoire de Physiologie et d’Explorations Fonctionnelles, Unité de Formation et de Recherche en Sciences Médicales, Université Félix Houphouët-Boigny, Abidjan, Côte d’Ivoire; ^2^Service des Explorations Fonctionnelles, Centre Hospitalier Universitaire de Yopougon, Abidjan, Côte d’Ivoire; ^3^Laboratoire de Biochimie, Unité de Formation et de Recherche en Sciences Pharmaceutiques et Biologiques, Université Félix Houphouët-Boigny, Abidjan, Côte d’Ivoire; ^4^Olopam Pharma and Research & Development, Abidjan, Côte d’Ivoire; ^5^Service des Explorations Fonctionnelles, Centre Hospitalier Universitaire de Cocody, Abidjan, Côte d’Ivoire

**Keywords:** cocoa, blood pressure, heart rate, young men, Black African, flavanols cocoa and arterial blood pressure

## Abstract

**Background:**

In Caucasians, regular consumption of cocoa induces a drop in arterial blood pressure via an increase in nitric oxide (NO) production. However, black individuals have a different NO biodisponibility compared to Caucasians. The aim of this study was to determine, in black Africans, the physiological variations in arterial blood pressure among cocoa consumers.

**Method:**

In total, 49 male black African volunteers, aged between 18 and 30 years old, were randomized into two groups; those consuming 10 g of cocoa powder per day (1,680 mg of flavonoids per day) for 3 weeks (consumer group), and those not consuming cocoa (control group). Systolic (SBP) and diastolic blood pressures (DBP), and heart rate (HR) were measured in the morning on an empty stomach (fasting), on day (D) 1 (without cocoa), D8, D15, and D22. Data were collected by groups and by subgroups established according to the level of SBP, DBP, or HR on D1. The means and variations of the means (between D1 and the subsequent days) of the recorded parameters were calculated and compared between groups and between subgroups.

**Results:**

On D8, the variations in SBP in the consumer group were significantly different from the control group (−3.72 ± 6.01 versus 0.57 ± 6.66 mmHg; *p* = 0.02). Between the control and consumer subgroups according to SBP, no statistical difference in the means or variations in SBP was noted. On D8 and D22, the variations in the SBP of consumers with SBP ≥ 110 mmHg on D1 were significantly different from those of other consumers (D8: −6.55 ± 5.96 versus −1.1 ± 4.93 mmHg; *p* = 0.01; D22: −6.63 ± 7.77 versus 0.35 ± 5.58 mmHg; *p* = 0.01). In the subgroups with a DBP < 75 mmHg on D1, the mean DBP of the consumers was significantly lower than that of the controls on D8 (65 ± 5 versus 69 ± 6 mmHg; *p* = 0.03).

**Conclusion:**

In young black African men living in Côte d’Ivoire, regular consumption of cocoa resulted in a decrease in SBP and DBP. The decrease in SBP appeared to be greater the higher the baseline SBP was.

## Introduction

High blood pressure is a public health problem around the world (Ncd Risk Factor Collaboration (Ncd-RisC), 2017). According to the World Health Organization (WHO), high blood pressure-related cardiovascular complications are the cause of 9.4 million deaths worldwide (Lim et al., 2012; Organisation Mondiale de la Santé Genève (OMS), 2011). It is estimated that by 2025, 1.56 billion adults worldwide will have high blood pressure (Kearney et al., 2005). In Africa, arterial hypertension affects young people, mostly between 31 and 55 years old (Campia et al., 2004; Hendriks et al., 2012; Ataklte et al., 2015; Desormais et al., 2019). A study on a population of healthy young black African adults aged 18 to 30 years showed an increase in both systolic (SBP) and diastolic blood pressures (DBP) with age (Siransy-Balayssac et al., 2020), with the risk of complications and mortality increasing with increasing blood pressure (Society of Actuaries, 1959; Arteriosclerosis, 1978). By 2025, it is estimated that more than 150 million people in Africa will have high blood pressure compared to 80 million in 2000 (Yaya and Kengne, 2014). The prevention of this scourge is therefore a priority.

For many years, to prevent an abnormal rise in blood pressure, the learned cardiology societies have recommended some hygieno-dietetic measures such as the regular consumption of fruits and vegetables as a first line of treatment (Dzudie et al., 2017; Bryan et al., 2018; Nerenberg et al., 2018; Unger et al., 2020). Several studies have highlighted the effects of cocoa flavanols (organic molecules of the polyphenol family) on blood pressure (Heiss et al., 2003; Keen et al., 2005; Taubert et al., 2007a,b; Ried et al., 2010, 2017; Fraga and Oteiza, 2011). These flavanols are believed to increase the production of nitric oxide (NO) by endothelial cells, which has a vasodilating effect and leads to a decrease in blood pressure (Taubert et al., 2007a). Studies on the effects of regular cocoa consumption on blood pressure have been carried out starting from the consumption of 3.6–105 g of cocoa (30–1,218 mg of flavanols) per day, over a period of 2–18 weeks (Ried et al., 2010, 2017; Hauhouot-Attoungbré et al., 2011; Massee et al., 2015; Balayssac-Siransy et al., 2018). The compiled results of these studies have highlighted a difference between the blood pressures of cocoa consumers and those of controls. Based on all levels of arterial pressure taken together at entry into the study, this difference was between −5.08 and −0.43 mmHg for SBP, and between −2.57 and −0.69 mmHg for DBP, reflecting a drop in pressure among consumers. However, only some studies identified a decrease in arterial pressure (Grassi et al., 2005; Crews et al., 2008; Davison et al., 2008; Shiina et al., 2009; Fraga and Oteiza, 2011), while others identified an increase (Murphy et al., 2003; Engler et al., 2004; Njike et al., 2011; Massee et al., 2015). These conflicting results may be due to several factors, including the amount of flavanols consumed and the characteristics of the study population (Huxley and Neil, 2003; Minor et al., 2008; Ried et al., 2017).

Regarding the study population, these previous studies were mainly carried out in mixed gender populations (Ried et al., 2010, 2017), even though some variations in blood pressure have been identified with different phases of the menstrual cycle (Moran et al., 2000; Balayssac-Siransy et al., 2014). Several studies have revealed a greater decrease in SBP and DBP in subjects under 50 years of age (Ried et al., 2010, 2017). Finally, these studies were mainly carried out in Caucasian populations (Desch et al., 2010; Ried et al., 2010, 2017), while various studies have pointed out low levels of NO bioavailability as well as low levels of its precursor, L-Arginine, in black subjects compared to Caucasians (Kalinowski et al., 2004; Glyn et al., 2012; Morris et al., 2013; Ozkor et al., 2014). This low level of NO bioavailability in black subjects could reduce the effects of cocoa flavanols on the vascular endothelium, subsequently leading to no change or a small decrease in arterial blood pressure. Available studies on black African participants are rare. A previous study carried out by our team in a population of young, healthy, male, black Africans did not find a significant difference in the reduction in blood pressure between those who received 5 g of 100% cocoa powder per day (1,475 mg of polyphenols, 840 mg of flavonoids) for 3 weeks and controls who did not consume any cocoa (Balayssac-Siransy et al., 2018). We considered that this dose of flavonoids may have been insufficient. Thus, the aim of the present study was to determine the weekly variations in SBP and DBP of young black African males consuming 10 g of 100% cocoa powder (2,950 mg of polyphenols, 1,680 mg of flavonoids) over 3 weeks.

## Materials and Methods

### Ethics Approval

This study was approved by the Ethics Committee of the University Teaching Hospital of Yopougon (Abidjan, Côte d’Ivoire) and followed the guidelines of the Declaration of Helsinki. All patients were informed on the purpose of the study protocol and gave their written consent.

### Study Population

#### Target Population

The population consisted of volunteer students from both the Félix Houphouët-Boigny and Nangui Abrogoua universities (Abidjan, Côte d’Ivoire).

#### Selection Criteria

Those included in the study were of black African origin, male, aged between 18 and 30 years, and had a body mass index (BMI) between 18.5 and 29.9 kg/m^2^. Non-inclusion criteria were smoking, regular alcohol consumption, diabetes, dyslipidemia, a cardiovascular (including arterial hypertension), respiratory, hematological or infectious symptomatology or disease, unexplained fatigue, a physical activity score greater than 35 points (corresponding to very active subjects) according to Ricci and Gagnon scoring system, recent or current intake of a drugs that can modify the blood pressure or heart rate (HR), and regular consumption of all cocoa-based products or products rich in flavonoids (fruits, nuts, coffee, tea, and wine).

#### Completion of the Survey Form

‘These inclusion and non-inclusion criteria were assessed using a survey sheet organized into two parts: a questionnaire and a record of anthropometric measurements. The questionnaire portion was performed by the participant in the presence of an investigator. The height of participants was measured, without shoes, using a vertical measuring rod graduated in centimeters (Gima, Italy). Participants stood, knees straight and looking ahead, with their arms alongside their body, with their feet together and their heels against the measuring rod. The weight of participants was measured with an electronic scale (Exacta Type Premium, Germany). For measuring, participants wore light clothes, without a belt, and with empty pockets. The weight was displayed in kg with a margin of error of 0.1 kg.

#### Sample

**FIGURE 1:**
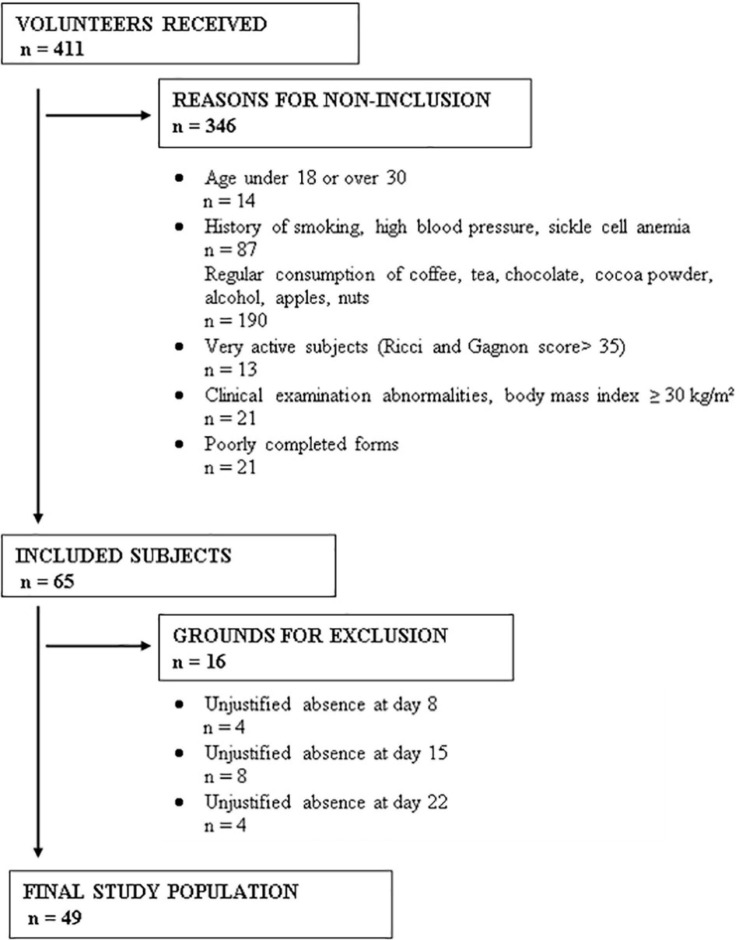
Reasons for non-inclusion and exclusion of subjects for this study.

Among 411 volunteer students, 65 were selected according to our selection criteria (Figure 1) and were then randomized into two groups: 32 in the control group and 33 in the cocoa consumer group. During the 3 weeks of the study, participants were excluded if their systolic blood pressure dropped lower than 90 mmHg, they took a drug that can modify blood pressure or HR, they were unable to attend the appointment, they withdrew from the study, they required medical intervention, they experienced major stress, they suffered from adverse effects linked to the consumption of cocoa powder (an allergy, digestive disorders, disgust, nervousness), or if they were absent without justification. Based on these exclusion criteria, 16 subjects were excluded (Figure 1). Therefore, 49 subjects fully completed the study protocol: 24 controls and 25 cocoa consumers, with a mean age of 21.4 ± 2.3 years and 22 ± 2.6 years, respectively (*p* = 0.47), and a mean BMI of 21.5 ± 2 kg/m^2^ and 21.6 ± 2.1 kg/m^2^, respectively (*p* = 0.94).

### Study Material

#### Equipment

An electronic scale (Exacta type Premium, Germany) was used for the weight measurement in kilograms. Electronic sphygmomanometers (Omron type M6, Japan) were used to measure SBP, DBP, and HR.

#### Cocoa Powder

Three brands of 100% cocoa powder available in supermarkets in Abidjan (Côte d’Ivoire) were selected: TAFISSA, NESTLE, and RYAN’S. The analysis of polyphenols and flavonoids in these powders were carried out by the Laboratory of Industrial Processes, Synthesis, Environment and New Energies (LAPISEN) of the National Polytechnic Institute Houphouët-Boigny of Yamoussoukro (Côte d’Ivoire). The method of Wood et al. (2002) was used for measuring total polyphenol content. Total flavonoid content was measured according to Marinova et al. (2005). Analyses indicated a higher flavonoid content in the TAFISSA and NESTLE powders. When taking into consideration the availability of a 5 g stick packaging, the ease of supply on the local market, and the cost of the powder, TAFISSA was retained for this study, which is a cocoa powder from the Forasteros variety (100% cocoa, nothing added). The sticks used came from the following three batches: PS100-17 074/17-088 from March 15, 2017 to March 15, 2019; PS100-17 075/17-131 from March 16, 2017 to March 16, 2019; PS100-17 138/17-143 from May 18, 2017 to May 18, 2019.

#### Sheets

A follow-up form filled out at each meeting made it possible for participants to notify us about the presence of acute stress, symptoms, or use of medications during the 7 days preceding the current visit. A food sheet given to each subject at the beginning of each week was used for the daily recording of the types of food consumed and for recording any medication taken. On this sheet, the instructions to be followed were provided, including foods to avoid and directing participants to consume cocoa in the morning on an empty stomach thirty minutes before breakfast.

#### Study Protocol

This was an experimental, prospective, randomized, single-blind study (the investigators at the measuring station were blinded to the grouping of the participant). The protocol was performed in two steps.

The subjects included in the study were informed of the foods to avoid for the 7 days before the date set for their entry into the 2nd stage. This included food products containing a significant amount of flavanols (foods made from cocoa, fruits, tea, coffee, nuts, wine, and other alcohols).

The 2nd stage lasted 3 weeks, during which participants were assessed four times: On day (D)1 (1st day, without cocoa), on D8 [8th day corresponding with the end of the 1st week (W1)], on D15 [15th day corresponding with the end of the 2nd week (W2)], and D22 [22nd day corresponding with the end of the 3rd week (W3)].

The follow-up protocol at this second stage was the same on each of the four visits. Participants were directed to fast for ten hours before arriving for assessment between 7 am and 8 am. An individual follow-up sheet looked for specific dates of major stress, symptoms, or drug intake. Body weight was measured as described previously and participants were then placed in groups of 3, in a seated position (back and arms supported with the feet on the ground). Each participant faced toward one of the corners of the quiet, semi-lit and air-conditioned room at 22°C. Blood pressure cuffs were placed on the non-dominant arm of each subject. After 5 min of rest, the arterial blood pressure was measured according to the recommendations of the ESH and ESC (Giuseppe et al., 2013). SBP and DBP arterial blood pressures and HR were recorded at the 5th, 7th, and 10th minutes of rest. On each of the 4 visits, each participant occupied the same place (previously numbered) in the same room and the measurements were made with the same blood pressure monitor (previously numbered).

After leaving the blood pressure measurement room, the participants were received, in turn, in another room by a single investigator appointed for the duration of the study. During the first visit (D1), this investigator communicated the study group (consumer or control) to each participant while specifying that this information should not be communicated to a third person (neither participant nor investigator). The investigator then diluted two sticks of 5 g of cocoa powder in water that the participant consumed in his presence. The investigator then gave the participant the 12 other sticks of cocoa powder for the remaining 6 days of the week with a food sheet to be completed by the participant. The control participants were also received individually like the consumer participants. They received neither cocoa nor a placebo and were given the same confidentiality instructions. Throughout the study, no participant had to change his usual level of physical activity or diet.

### Statistical Analysis Plan

#### Measured and Calculated Parameters

**FIGURE 2:**
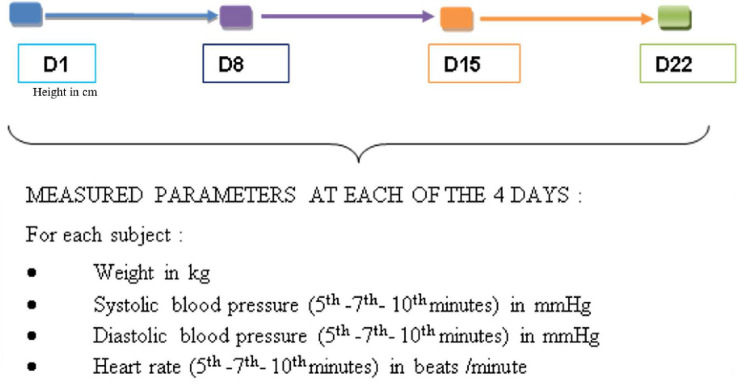
Parameters measured on each visit day.

**FIGURE 3:**
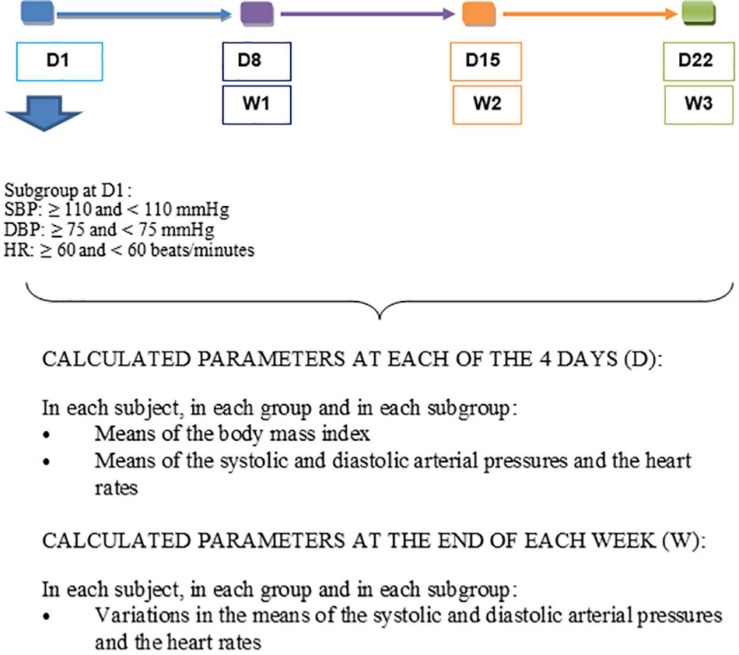
Parameters calculated at the end of each of the 3 weeks.

For each participant at each of the four visits, the individual averages of SBP, DBP, and HR were obtained from the three measurements taken at the 5th, 7th, and 10th minute at rest (Figure 2). In each group, the mean SBP, DBP, and HR were established from the individual means (Figure 3). From the data of D1, the subgroups were formed within the control and consumer groups on the basis of a previous study which focused on the regular consumption of 5 g of 100% cocoa powder in the same target population (Balayssac-Siransy et al., 2018): 110 mmHg for SBP, 75 mmHg for DBP, and 60 beats per minute for HR at D1 (Figure 3). In each subgroup, the mean SBP, DBP, and HR were calculated (Figure 3). The mean BMI of the groups and subgroups were calculated from the individual values taken of the height on D1 and the weight taken on the day of assessment. From the averages of SBP, DBP, and HR, the mean variations in SBP, DBP, and HR were calculated based on the D1values at each time point within the 3-week period (D8 or W1, D15 or W2, and D22 or W3) at an individual level, by group, and by subgroup (Figure 3). The comparisons of the means at D1, D8, D15, and D22, and the mean variations at W1, W2, and W3 were made within groups, within subgroups, between groups, and between subgroups.

#### Statistical Analyzes

The data obtained were imported, cleaned, categorized, and analyzed with R4.0.2 software. Since the distribution of groups and subgroups does not always follow a normal distribution, comparative analyzes of the means of groups and subgroups were made using the Wilcoxon–Mann–Whitney test for comparisons of means between the two groups or two subgroups, and Friedman’s test was applied to compare the weekly evolution of parameters over the 3 weeks in groups and subgroups. The tests evaluating the changes in parametric values over time were one-sided. Multiple linear regression was used to estimate the effect of cocoa consumption on changes in SBP, DBP, and HR. The analyses were carried out with a type I error of 0.05.

#### Sample Size Calculation

The sample size determination was based on the Student’s *t*-test formula for paired populations, to detect a change of at least 1 mmHg in arterial pressure in cocoa consumers with a standard deviation of 1.5 mmHg, taking into account a previous study in a similar population who consumed 5 g of TAFISSA cocoa powder for 3 weeks (Balayssac-Siransy et al., 2018). The minimum required size calculated was 21 subjects per group.

## Results

### Body Mass Index

During the 3-week follow-up, there were no significant differences in the means of the BMI in the controls, the consumers, or between these two groups (Table 1).

**TABLE 1:** Body mass index of control and consumer participants.

Groups	Means body mass index (kg/m^2^)				*p*
	D1	D8	D15	D22	
Control *n* = 24	21.5 ± 2	21.5 ± 1.9	21.4 ± 2	21.4 ± 2	0.66
Consumer *n* = 25	21.6 ± 2.1	21.7 ± 2	21.7 ± 2.1	21.7 ± 2.1	0.83
*p*	0.94	0.93	0.10	0.88	

*Data expressed as mean ± standard deviation. D1: basic measurement without cocoa intake on the first day; D8: eighth day; D15: fifteenth day; D22: twenty second day; *n*: number of participants; *p*: value of *p* (significant if *p* < 0.05).*

### Systolic Blood Pressure

During the 3 weeks, there was no significant difference in mean SBP between the controls and consumers (Table 2). The variation in mean SBP in the cocoa consumer group was significantly different from that of the control group during the 1st week of follow-up (W1) (Table 2). Multiple linear regression made it possible to note that an increase of 1 mmHg in SBP at D1 lead to a significant decrease of 3.76 mmHg in SBP at the end of the 1st week of follow-up among consumers in comparison with controls (*p* = 0.028). At the end of the 2nd (W2) and 3rd (W3) weeks, this decrease was 2.63 and 2.2 mmHg, respectively, without any statistical significance (*p* = 0.2 and *p* = 0.2, respectively). When comparing the subgroups, no statistical difference in the means or the mean variations was observed between the control and consumer subgroups with an SBP at D1 < 110 mmHg (Table 3), or between those with an SBP at D1 greater ≥ 110 mmHg (Table 3).

**TABLE 2:** Means and means’ variations in systolic blood pressure of control and consumer participants.

Groups	Systolic blood pressure (mmHg)								
	Means				*p*	Variations of the means			*p*
	D1	D8	D15	D22		W1	W2	W3	
Controls *n* = 24	110 ± 8	110 ± 8	109 ± 9	110 ± 9	0.22	0.57 ± 6.66	−1.08 ± 6.18	−0.13 ± 6.07	0.13
Consumers *‘’n* = 25	112 ± 11	108 ± 10	107 ± 11	109 ± 9	0.05	−3.72 ± 6.01	−4.16 ± 8.36	−3 ± 7.48	0.47
*p*	0.51	0.14	0.33	0.53		0.02	0.13	0.09	

*Data expressed as mean ± standard deviation. D1: basic measurement without cocoa intake on the first day; D8: eighth day; D15: fifteenth day; D22: twenty second day; W1: end of the 1st week; W2: end of the 2nd week; W3: end of the 3rd week; *n*: number of participants; *p*: value of *p* (significant if *p* < 0.05).*

**TABLE 3:** Means and means’ variations in systolic blood pressure of the control and consumer subgroups based on systolic blood pressure at D1.

Subgroups with SBPS_*J1*_ < 110 mmHg	Systolic Blood pressure *(mmHg)*								
	Means				*p*	Variations of the means			*p*
	D1	D8	D15	D22		W1	W2	W3	
Controls *n* = 14	104 ± 4	107 ± 4	104 ± 4	105 ± 4	0.44	2.94 ± 4.72	0.35 ± 5.2	1.47 ± 5.43	0.16
Consumers *n* = 13	104 ± 5	103 ± 6	103 ± 6	105 ± 6	0.62	−1.10 ± 4.93	−1.43 ± 6.70	0.35 ± 5.58	0.45
*P*	0.61	0.09	0.43	0.56		0.10	0.45	0.42	

**Subgroups with SBP_*J1*_ ≥ 110 mmHg**	**Means**				***p***	**Variations of the means**			***p***
	**D1**	**D8**	**D15**	**D22**		**W1**	**W2**	**W3**	

Controls *n* = 10	118 ± 6	115 ± 10	115 ± 11	115 ± 10	0.14	−2.73 ± 7.77	−3.10 ± 7.04	−2.40 ± 6.46	0.61
Consumers *n* = 12	119 ± 10	113 ± 11	112 ± 13	113 ± 9	0.06	−6.55 ± 5.96	−7.11 ± 9.24	−6.63 ± 7.77	0.55
*p*	0.86	0.25	0.17	0.29		0.35	0.40	0.17	

*Data expressed as mean ± standard deviation. D1: basic measurement without cocoa intake at the first day; D8: eighth day; D15: fifteenth day; D22: twenty second day; W1: end of the 1st week; W2: end of the 2nd week; W3: end of the 3rd week; *n*: number of participants; *p*: value of *p* (significant if *p* < 0.05).*

Among consumers, at the end of the 1st (W1) and 3rd (W3) weeks of follow-up, the negative variations in SBP (reflecting a decrease in SBP) in the consumer subgroup with an SBP at D1 ≥ 110 mmHg were significantly greater than the variations of the consumer subgroup with an SBP at D1 ≤ 110 mmHg (Table 4). However, no statistical difference was observed in the variations of SBP between the two control subgroups based on the level of SBP at D1 (Table 4).

**TABLE 4:** Comparison of the mean variations in systolic blood pressure between the control and consumer subgroups based on systolic blood pressure at D1.

Subgroups	Variations of the means systolic blood pressure (mmHg)					
	Controls (*n* = 14/10)			Consumers (*n* = 13/12)		
	W1	W2	W3	W1	W2	W3
SBP_*J1*_ < 110 mmHg	2.94 ± 4.72	0.35 ± 5.2	1.47 ± 5.43	−1.10 ± 4.93	−1.43 ± 6.70	0.35 ± 5.58
SBP_*J1*_ ≥ 110 mmHg	−2.73 ± 7.77	−3.10 ± 7.04	−2.40 ± 6.46	−6.55 ± 5.96	−7.11 ± 9.24	−6.63 ± 7.77
*p*	0.09	0.43	0.56	0.01	0.14	0.01

*Data expressed as mean ± standard deviation. W1: end of the 1st week; W2: end of the 2nd week; W3: end of the 3rd week; *n*: number of participants; *p*: value of *p* (significant if *p* < 0.05).*

### Diastolic Blood Pressure

During the follow-up period, there were no significant changes in the mean or the mean variations of DBP in the control and consumer groups (Table 5). The mean DBP of the control group was significantly higher than that of the consumer group from D1 to D15 (Table 5), but no statistical difference was noted in the mean variations of the DBP during the follow-up period between these two groups (Table 5).

**TABLE 5:** Means and means’ variations of the diastolic blood pressure of control and consumer participants.

Subgroups with DBP_*J1*_ < 75 mmHg	Diastolic blood pressure (mmHg)								
	Means				*p*	Variations of the Means			*p*
	D1	D8	D15	D22		W1	W2	W3	
Controls *n* = 13	68 ± 5	69 ± 6	69 ± 6	68 ± 6	0.89	0.94 ± 4.15	0.61 ± 4.61	−0.30 ± 6.15	0.87
Consumers *n* = 21	67 ± 4	65 ± 5	65 ± 5	67 ± 4	0.45	−1.77 ± 5.41	−1.66 ± 5.44	−0.17 ± 4.40	0.19
*p*	0.30	0.03	0.07	0.34		0.19	0.30	0.84	

**Subgroups with DBP_*J1*_ ≥ 75 mmHg**	**Means**				***p***	**Variations of the Means**			***p***
	**D1**	**D8**	**D15**	**D22**		**W1**	**W2**	**W3**	

Controls *n* = 11	77 ± 3	72 ± 4	72 ± 4	72 ± 4	0.001	−4.86 ± 3.99	−5.72 ± 3.98	−4.97 ± 4.87	0.69
Consumers *n* = 4	84 ± 7	79 ± 6	76 ± 9	76 ± 4	0.22	−5.41 ± 7.36	−8.41 ± 11.25	−8.41 ± 7.23	0.62
*p*	0.10	0.05	0.43	0.55		0.94	1	0.64	

*Data expressed as mean ± standard deviation. D1: basic measurement without cocoa intake at the first day; D8: eighth day; D15: fifteenth day; D22: twenty second day; W1: end of the 1st week; W2: end of the 2nd week; W3: end of the 3rd week; *n*: number of participants; *p*: value of *p* (significant if *p* < 0.05).*

With the multiple linear regression analysis, the increase in DBP of 1 mmHg at D1 resulted in a decrease in the DBP of 1.68, 1.74, and 0.42 mmHg, respectively at the end of the 1st week (W1), the 2nd week (W2), and the 3rd week (W3) of follow-up in the consumers in comparison with control subjects, with no significant difference (*p* = 0.21, 0.24, and 0.74).

When comparing the two subgroups with a DBP < 75 mmHg at D1, the consumer subgroup had a significantly lower mean DBP than that of the corresponding control subgroup at D8 (Table 6), with no statistical difference in the mean variations of DBP between these two subgroups (Table 6). No statistical differences in the mean or the mean variations of DBP were observed between the control and consumer subgroups with a DBP ≥ 75 mmHg at D1 (Table 6).

**TABLE 6:** Means and means’ variations of the diastolic blood pressure of the controls and consumer subgroups based on diastolic blood pressure at D1.

Groups	Diastolic blood pressure (mmHg)								
	Means				*p*	Variations of the Means			*p*
	D1	D8	D15	D22		W1	W2	W3	
Controls *n* = 24	73 ± 6	71 ± 6	70 ± 5	70 ± 6	0.19	−1.71 ± 4.96	−2.29 ± 5.33	−2.44 ± 5.97	0.96
Consumers *n* = 25	70 ± 8	68 ± 7	67 ± 7	68 ± 6	0.45	−2.36 ± 5.75	−2.74 ± 6.85	−1.49 ± 5.67	0.41
*p*	0.017	0.017	0.02	0.08		0.50	0.65	0.77	

*Data expressed as mean ± standard deviation. D1: basic measurement without cocoa intake at the first day; D8: eighth day; D15: fifteenth day; D22: twenty-second day; W1: end of the 1st week; W2: end of the 2nd week; W3: end of the 3rd week; *n*: number of participants; *p*: value of *p* (significant if *p* < 0.05).*

### Heart Rate

During the 4 days of follow-up, comparison of the means and the mean variations in HR between the control and consumer groups, and in each of these two groups, did not find any statistical differences (Table 7).

**TABLE 7:** Means and means’ variations of the heart rate of the control and the consumer subjects.

Groups	Heart rate (beats per minute)								
	Means				*p*	Variations of the means			*p*
	D1	D8	D15	D22		W1	W2	W3	
Controls *n* = 24	70 ± 7	67 ± 9	67 ± 9	68 ± 12	0.51	−3.16 ± 7.67	−2.57 ± 8.35	−2.29 ± 11.94	0.95
Consumers *n* = 25	66 ± 9	64 ± 11	64 ± 9	63 ± 8	0.23	−1.73 ± 6.13	−1.98 ± 5.82	−2.68 ± 8.01	0.80
*p*	0.15	0.41	0.21	0.23		0.76	0.66	0.49	

*Data expressed as mean ± standard deviation. D1: basic measurement without cocoa intake at the first day; D8: eighth day; D15: fifteenth day; D22: twenty second day; W1: end of the 1st week; W2: end of the 2nd week; W3: end of the 3rd week; *n*: number of participants; *p*: value of *p* (significant if *p* < 0.05).*

## Discussion

This experimental, prospective, single-blind study was carried out over three weeks in healthy black African young males aged 18–30. It aimed to determine the weekly variations in systolic and diastolic arterial blood pressures of subjects consuming 10 g of 100% cocoa powder (2,950 mg of polyphenols, 1,680 mg of flavonoids) compared to those of control subjects not receiving any form of cocoa. This work demonstrated significantly greater weekly reductions in SBP and DBP in consumers than in controls. In the consumer group, the reductions in SBP were significantly greater among those with the highest SBP at entry into the study. This observation was not found in the control group.

### Systolic Blood Pressure

The comparison of the variations in SBP between the cocoa consumers and the controls found a greater decrease in SBP in consumers during the 3 weeks, with a significant difference only at the end of the 1st week. Likewise, multiple linear regression analysis showed that the increase in SBP of 1 mmHg at D1 was accompanied by a greater decrease in SBP in the consumer group compared to the control group at each time point over the 3 weeks, with statistical significance only at the end of the 1st week. In an earlier study (Balayssac-Siransy et al., 2018) conducted over 3 weeks in 49 male black African subjects aged 18–30 years, the weekly decreases in SBP in the 26 consumers of 5 g of 100% cocoa powder per day (1,475 mg polyphenols, 840 mg flavonoids, TAFISSA brand) were not statistically different from those of the 24 control subjects who received no form of cocoa. These results could be explained by the smaller quantity of flavonoids present in the 5 g of cocoa powder compared to the 10 g in the present study (2,950 mg of polyphenols, 1,680 mg of flavonoids, TAFISSA brand). On the other hand, our results concur with those of Grassi et al. (2005) who, in normotensive Caucasian subjects, also noted a significantly greater decrease in SBP in consumers receiving 100 g of dark chocolate (88 mg of flavanols) per day compared to the controls receiving 90 g of white chocolate per day (0 mg of flavanols) for 15 days. The decreases in SBP observed during the consumption of cocoa are thought to be linked to the action of cocoa flavanols on the vascular endothelium. Flavanols stimulate the endothelial enzyme, NO-synthase, thereby promoting an increased endothelial production of NO, which causes vasodilation and a consequent drop in blood pressure. In addition, the flavanols in cocoa might inhibit angiotensin I converting enzyme and therefore prevent formation of angiotensin II, a powerful vasoconstrictor. Alternatively, cocoa flavanols may inhibit the vasoconstrictor action of endothelin, thus causing a drop in blood pressure (Lamuela-Raventos et al., 2005; Ried et al., 2017). The existence of significative differences between consumers and controls could be linked to the amount of polyphenols, flavonoids, or flavanols contained in the consumed product. In fact, the decrease in SBP is relative to the quantity of flavanols consumed (Al-Faris, 2008). The individual variability in the response to the flavanols could play a role and make the SBP variations heterogeneous. Weekly dosages of NO, endothelin-1, and converting enzyme metabolites in the consumers may help to understand the variations and possibly to classify the individuals.

In our population of normotensive black African subjects, a weekly reduction in the consumer SBP (between −4.16 and −3 mmHg) was included within the range of SBP variations in the studies conducted in black normotensive consumers (Hauhouot-Attoungbré et al., 2011; Balayssac-Siransy et al., 2018) and Caucasians (Desch et al., 2010; Ried et al., 2010; Massee et al., 2015). Indeed, in these studies, the variations in the SBP were between −7 and 4.6 mmHg after a daily consumption of cocoa in the form of chocolate or cocoa powder, sweet or not sweet, over a period of 2–18 weeks. Apart from the amount of flavanols consumed, the negative (decreases) or positive (increases) variations in the SBP among consumers, from one study to the next, are thought to be linked to various factors. The simultaneous consumption of cocoa and 10 g of sugar would cancel out the endothelial vasodilator effects of the flavanols (Ried et al., 2017). In addition, changes in diet and the level of physical activity during monitoring would influence the SBP values. Consuming cocoa with a meal may reduce the absorption and therefore the effects of flavanols. The presence of confounding factors such as stress or taking medication might modify the values of the SBP.

Taking into account the level of SBP at D1 (<110 mmHg or ≥110 mmHg), there was no statistical difference between the weekly variations in SBP of the consumers (1,680 mg flavonoids/day) and those of the controls, with both groups having the same SBP at D1. These results were identical to those of Balayssac-Siransy et al. (2018) using 26 consumer subjects (840 mg flavonoids/day) and 24 controls. The lack of difference in SBP between the control and consumer groups could be due to the use of normotensive participants in this study. Indeed, the reductions in the SBP have been shown to be less significant in normotensive consumers than in prehypertensive and hypertensive consumers, with the drawback of smaller differences with the controls (Desch et al., 2010; Ried et al., 2010, 2017).

In our group of cocoa consumers, the decreases in the SBP of those with an SBP ≥ 110 mmHg at D1 were statistically greater than those of consumers at D1 with a lower SBP (<110 mmHg) at W1 and W3 of follow-up. These statistical differences were not noted in the control group. These results, depending on the level of SBP of the consumers, were found in other studies carried out in black (Balayssac-Siransy et al., 2018) or Caucasian (Desch et al., 2010; Ried et al., 2010) populations. The variations in SBP in consumers with a low baseline blood pressure ranged from −7 to 4.6 mmHg versus −12 to −2 mmHg in those with a higher baseline SBP. The greater decrease in SBP observed with the baseline level of SBP is believed to be due to the fact that the higher the baseline SBP, the more the NO production is increased via greater stimulation of NO synthase (Taubert et al., 2007a).

### Diastolic Blood Pressure

The comparison of the DBP between the consumers and controls revealed a statistical difference in the means of the DBP from D1 (baseline value before any cocoa consumption) but without statistical difference in the mean variations over the 3 weeks of follow-up. The consumers with a DBP < 75 mmHg at D1 exhibited a significantly lower DBP than the controls with the same baseline level of DBP at D8.

Some non-significant variations in the DBP between consumers and controls were also found by Balayssac-Siransy et al. (2018) in normotensive black subjects in which the 26 consumers had received 5 g of 100% cocoa powder, and the 24 controls received no form of cocoa. In this same population, no difference in the mean variations in DBP was noted between consumers and controls with the same baseline level of DBP. Similarly, non-significant variations in DBP between consumers and controls were reported by Crews et al. (2008) after 6 weeks of follow-up of 45 normotensive Caucasians who consumed chocolate and cocoa drinks (37 g/day and 237 ml/day) and 45 controls who did not consume cocoa (D15: −0.5 ± 6.06 versus −0.57 ± 7.15 with *p* = 0.98). On the other hand, a study by Grassi et al. (2005) involving normotensive Caucasians demonstrated a significant decrease in DBP of consumers of dark chocolate (100 g/day containing 88 mg of flavanols) compared to controls receiving white chocolate not containing any flavanols. These differences could be multifactorial in that they may be affected by the product consumed, quantity of flavanols, measurement protocol, and individual response. A multicenter study with the same methodology would be helpful in understanding these results.

In the consumer group, the weekly decreases in DBP obtained during the 3 weeks of follow-up (between −2.74 and −1.49 mmHg) were comparable to those found in the Caucasian populations (Desch et al., 2010; Ried et al., 2010; Heiss et al., 2015). In fact, in these populations, the variations in DBP were between −7 and 6.6 mmHg after daily consumption of cocoa over 2–18 weeks. When consuming cocoa, the flavanols would lead to an increase in NO production and vasodilation by acting on the vascular endothelium (Sudano et al., 2012), with a drop in blood pressure as a drawback. They might also be responsible for a decrease in arterial stiffness and a decrease in the peripheral resistance (Ana et al., 2016), leading to a drop in blood pressure.

### Heart Rate

The present study did not find a statistical difference between the reductions in HR of the control subjects and those of cocoa consumers. The same was observed amongst the subgroups of the controls and the cocoa consumers according to the HR at baseline. These results are in agreement with those of Balayssac-Siransy et al. (2018) investigating a population of black Africans in which the consumers only received cocoa (840 mg of flavonoids per day). In contrast, Crews et al. (2008) conducted a study involving 90 Caucasians comprising 45 consumers of chocolate (37 g/day) and cocoa drinks (237 ml/day), and 45 controls that did not consume cocoa. After 6 weeks of follow-up, a significant increase in HR was noted in the consumers compared with the controls (4.72 ± 7.93 versus 0.33 ± 8.19 beats/minute, *p* = 0.007). The differences between these studies could be explained by the quantity of cocoa received. As cocoa is rich in minerals, including potassium, a study focusing on the relationship between the amount of potassium in cocoa and HR variations would help form a better understanding of the variations in HR when consuming cocoa.

Among consumers of the present study, no statistical difference was noted according to the HR at D1. These results were not in agreement with those of Balayssac-Siransy et al. (2018), who investigated 26 cocoa consumers (840 mg of flavonoids/day for 3 weeks) that demonstrated a significant decrease in HR in comparison with the controls in those with a HR ≥ 60 bpm at D1 during the 3 weeks of follow-up. A study based on the individual variability of HR in our population of black African origin could help to explain these different results.

### Limitations

Taking into account the genetic and environmental aspects of the populations, our results cannot be extrapolated to black Africans in general. Studies comparing black African populations will be needed in the future.

The selection of males only for this study was due to the fact that hormonal fluctuations in women during the menstrual cycle might influence the arterial blood pressure (Moran et al., 2000; Balayssac-Siransy et al., 2014). Therefore, to investigate the effects of cocoa on the cardiovascular systems of females, the study protocol should be different and should take place during the menstrual phases of two successive cycles.

Choosing participants aged 18–30 was motivated by the data in the literature, according to which the drop in the systolic and the diastolic arterial blood pressures induced by cocoa consumption would be greater in subjects under 50 years of age (Taubert et al., 2007b; Ried et al., 2017).

The single-blind study design could constitute a bias because it tends to induce a greater reduction in arterial blood pressures compared to double-blind studies, although not statistically significant according to the data in the literature (Ried et al., 2017).

We did not perform blood tests to measure the metabolites of NO nor the phenols of the cocoa or their metabolites responsible for stimulating the NO production and lowering the blood pressure during consumption of cocoa powder. The results of this work will allow us to program the dosages of NO in another study.

## Conclusion

In our population of young male, normotensive, black African subjects living in Côte d’Ivoire (West Africa), the daily consumption of 10 g of 100% cocoa powder (1,680 mg of flavonoids/day) demonstrated some significantly greater weekly reductions in SBP and DBP in the consumer group compared to the control group. Additionally, the drop in SBP in the consumer group increased with the baseline SBP unlike in the control group. In view of these results, a study focusing on the black male and female population, investigating regular consumption of cocoa and performing blood analysis to assess NO metabolites, converting enzyme, endothelin-1, and cocoa flavanols would make it possible to establish the mechanisms behind the lowering of arterial blood pressure in this black African population.

## Data Availability Statement

The original contributions presented in the study are included in the article/supplementary material, further inquiries can be directed to the corresponding author/s.

## Ethics Statement

This study was approved by the Ethics Committee of the University Teaching Hospital of Yopougon (Abidjan, Côte d’Ivoire) and followed the guidelines of the Declaration of Helsinki. All patients were informed on the purpose of the study protocol and gave their written consent.

## Author Contributions

ES-B and SO contributed to the conception of the study, data analysis and interpretation, drafting of the manuscript, and approval of the final version of the manuscript, and agreed to be accountable for all aspects of the work. HA involved in approval of the final version of the manuscript and agreed to be accountable for all aspects of the work. TY, FG, and KY involved in data acquisition and agreed to be accountable for all aspects of the work. ME involved in drafting of the manuscript and agreed to be accountable for all aspects of the work. CD involved in revising the manuscript critically and approval of the final version of the manuscript, and agreed to be accountable for all aspects of the work. PB involved in revising the manuscript critically, drafting of the manuscript, and approval of the final version of the manuscript, and agreed to be accountable for all aspects of the work. All authors contributed to the article and approved the submitted version.

## Conflict of Interest

The authors declare that the research was conducted in the absence of any commercial or financial relationships that could be construed as a potential conflict of interest.

## References

[B1] Al-Faris N. A. (2008). Short term consumption of a dark chocolate containing flavanols is followed by a significant decrease in normotensive population. *Pak. J. Nutr.* 7 773–781. 10.3923/pjn.2008.773.781

[B2] Ana C. A. Anca M. Adriana T. Vlad S. Irina-Iuliana C. (2016). The Cardiovascular effects of cocoa polyphenols: an overview. *Diseases* 4:39. 10.3390/diseases4040039 28933419 PMC5456324

[B3] Arteriosclerosis (1978). *Report of the 1977 Working Group to Review the 1971 Report by the National Heart and Lung Institute Task Force on Arteriosclerosis.* DHEW Report (NIH) 78-1526. Washington, DC: US Government Printing Office, 9.

[B4] Ataklte F. Erqou S. Kaptoge S. Taye B. Echouffo-Tcheugui J. B. Kengne A. P. (2015). Burden of undiagnosed hypertension in Sub-Saharan Africa: a systematic review and meta-analysis. *Hypertension* 65 291–298. 10.1161/hypertensionaha.114.04394 25385758

[B5] Balayssac-Siransy A. E. Adoubi A. Kouamé A. Sall F. Kouamé C. Y. Ouattara S. (2014). Cycle menstruel et paramètres hémodynamiques au repos chez la jeune femme noire africaine (Menstrual cycle and hemodynamic parameters at rest in young black African women). *Afr. Bioméd.* 19 8–18. 10.1016/j.sagf.2011.01.003

[B6] Balayssac-Siransy E. Ouattara S. Kouame B. A. Brou M. Kouame I. Boka K. J. M. (2018). Effets de la consommation régulière de 5g de poudre de cacao sur la pression artérielle de sujets noirs africains. (Effects of regular consumption of 5g of cocoa powder on the blood pressure of black African subjects). *Rev. Bio Afr.* 18 23–29.

[B7] Bryan W. Giuseppe M. Wilko S. Enrico A. R. Michel A. Michel B. (2018). 2018 ESC/ESH guidelines for the management of arterial hypertension the task force for the management of arterial hypertension of the European Society of Cardiology and the European Society of Hypertension. *J. Hypertens.* 36 1953–2041. 30234752 10.1097/HJH.0000000000001940

[B8] Campia U. Cardillo C. Panza J. A. (2004). Ethnic differences in the vasoconstrictor activity of endogenous endothelin-1 in hypertensive patients. *Circulation* 109 3191–3195. 10.1161/01.cir.0000130590.24107.d3 15148269

[B9] Crews W. D. Jr. Harrison D. W. Wright J. W. (2008). A double-blind, placebo-controlled, randomized trial of the effects of dark chocolate and cocoa on variables associated with neuropsychological functioning and cardiovascular health: clinical findings from a sample of healthy, cognitively intact older adults. *Am. J. Clin. Nutr.* 87 872–880. 10.1093/ajcn/87.4.872 18400709

[B10] Davison K. Coates A. M. Buckley J. D. Howe P. R. (2008). Effect of cocoa flavanols and exercise on cardiometabolic risk factors in overweight and obese subjects. *Int. J. Obes. Lond.* 32 1289–1296. 10.1038/ijo.2008.66 18504447

[B11] Desch S. Schmidt J. Kobler D. Sonnabend M. Eitel I. Sareban M. (2010). Effect of cocoa products on blood pressure: systematic review and meta-analysis. *Am. J. Hypertens.* 23 97–103. 10.1038/ajh.2009.213 19910929

[B12] Desormais I. Amidou S. A. Houehanou Y. C. Houinato S. D. Gbagouidi G. N. Preux P. M. (2019). The prevalence, awareness, management and control of hypertension in men and women in Benin, West Africa: the TAHES study. *BMC Cardiovasc. Disord.* 19:303. 10.1186/s12872-019-01273-7 31881946 PMC6933658

[B13] Dzudie A. Rayner B. Ojji D. Aletta E. Twagirumukiza M. Damasceno A. (2017). Roadmap to achieve 25% hypertension control in Africa by 2025. *Cardiovasc. J. Afr.* 28 262–273. 10.5830/CVJA-2017-040 28906541 PMC5642030

[B14] Engler M. B. Engler M. M. Chen C. Y. Malloy M. J. Browne A. Chiu E. Y. (2004). Flavonoid-rich dark chocolate improves endothelial function and increases plasma epicatechin concentrations in healthy adults. *J. Am. Coll. Nutr.* 23 197–204. 10.1080/07315724.2004.10719361 15190043

[B15] Fraga C. G. Oteiza P. I. (2011). Dietary Flavonoids: role of (-) epicatechin and related procyanidins in cell signaling. *Free Radic. Biol. Med.* 51 813–823. 10.1016/j.freeradbiomed.2011.06.002 21699974

[B16] Giuseppe M. Robert F. Krzysztof N. Josep R. Alberto Z. Michael B. (2013). 2013 ESH/ESC Guidelines for the management of arterial hypertension: the Task Force for the management of arterial hypertension of the European Society of Hypertension (ESH) and of the European Society of Cardiology (ESC). *Eur. Heart J.* 34 2159–2219. 10.1093/eurheartj/eht151 23771844

[B17] Glyn M. C. Anderssohn M. Lüneburg N. Van Rooyen J. M. Schutte R. Huisman H. W. (2012). Ethnicity-specific differences in L-arginine status in South African men. *J. Hum. Hypertens.* 26 737–743. 10.1038/jhh.2011.103 22129611

[B18] Grassi D. Lippi C. Necozione S. Desideri G. Ferri C. (2005). Short term administration of dark chocolate is followed by a significant increase in insulin sensitivity and a decrease in blood pressure in healthy persons. *Am. J. Clin. Nutr.* 81 611–614. 10.1093/ajcn/81.3.611 15755830

[B19] Hauhouot-Attoungbré M. L. Yayo S. E. Yao C. Aké-Edjeme A. Hugues A. Yapi H. F. (2011). Consumption of dark chocolate reduced lipoprotein cardiovascular risk factors in an Ivorian population. *Biochm. Clin.* 35 190–192.

[B20] Heiss C. Dejam A. Kleinbongard P. Schewe T. Sies H. Kelm M. (2003). Vascular effects of cocoa rich in flavan-3-ols. *JAMA* 290 1030–1031. 12941674 10.1001/jama.290.8.1030

[B21] Heiss C. Sansone R. Karimi H. Krabbe M. Schuler D. Rodriguez-Mateos A. (2015). Impact of cocoa Flavanol intake on age-dependent vascular stiffness in healthy men: a randomized, controlled, double masked trial. *Age (Dordr.)* 37:9794. 10.1007/s11357-015-9794-9 26013912 PMC4444618

[B22] Hendriks M. E. Wit F. W. N. M. Roos M. T. L. Brewster L. M. Akande T. M. De Beer I. H. (2012). Hypertension in Sub-Saharan Africa: cross-sectional surveys in four rural and urban communities. *PLoS One* 7:e32638. 10.1371/journal.pone.0032638 22427857 PMC3299675

[B23] Huxley R. R. Neil H. A. (2003). The relation between dietary flavonol intake and coronary heart disease mortality: a meta-analysis of prospective cohort studies. *Eur. J. Clin. Nutr.* 57 904–908. 10.1038/sj.ejcn.1601624 12879084

[B24] Kalinowski L. Dobrucki I. T. Malinski T. (2004). Race-specific differences in endothelial function: predisposition of African Americans to vascular diseases. *Circulation* 109 2511–2517. 10.1161/01.cir.0000129087.81352.7a 15159296

[B25] Kearney P. M. Whelton M. Reynolds K. Muntner P. Whelton P. K. He J. (2005). Global burden of hypertension: analysis of worldwide data. *Lancet* 365 217–223. 10.1016/S0140-6736(05)17741-1 15652604

[B26] Keen C. L. Holt R. H. Oteiza P. I. Fraga C. G. Schmitz H. H. (2005). Cocoa antioxidants and cardiovascular health. *Am. J. Clin. Nutr.* 81 298S–303S. 15640494 10.1093/ajcn/81.1.298S

[B27] Lamuela-Raventos R. M. Romero-Perez A. I. Andres-Lacueva C. Tornero A. (2005). Health effects of cocoa flavonoids. *Food Sci. Technol. Int.* 113 159–176. 10.1177/1082013205054498

[B28] Lim S. S. Vos T. Flaxman A. D. Danaei G. Shibuya K. Adair-Rohani H. (2012). A comparative risk assessment of burden of disease and injury attributable to 67 risk factors and risk factor clusters in 21 regions, 1990-2010: a systematic analysis for the Global Burden of Disease Study 2010. *Lancet* 380 2224–2260. 10.1016/S0140-6736(12)61766-8 23245609 PMC4156511

[B29] Marinova D. Ribarova F. Atanassova M. (2005). Total phenolics and total flavonoids in Bulgarian fruits and vegetables. *J. Chem. Technol. Metall.* 40 255–260.

[B30] Massee L. A. Ried K. Pase M. Travica N. Yoganathan J. Scholey A. (2015). The acute and sub-chronic effects of cocoa Flavanols on mood, cognitive and cardiovascular health in young healthy adults: a randomized, controlled trial. *Front. Pharmacol.* 6:93. 10.3389/fphar.2015.00093 26042037 PMC4438591

[B31] Minor D. S. Wofford M. R. Jones D. W. (2008). Racial and ethnic differences in hypertension. *Curr. Atheroscler. Rep.* 10 121–127. 18417066 10.1007/s11883-008-0018-y

[B32] Moran V. H. Leathard H. Coley J. (2000). Cardiovascular functioning during the menstrual cycle. *Clin. Physiol.* 6 496–504. 10.1046/j.1365-2281.2000.00285.x 11100398

[B33] Morris A. A. Patel R. S. Binongo J. N. G. Poole J. al Mheid I. Ahmed Y. (2013). Racial Differences in arterial stiffness and microcirculatory function between black and white Americans. *J. Am. Heart Assoc.* 2:e002154. 10.1161/JAHA.112.002154 23568343 PMC3647269

[B34] Murphy K. J. Chronopoulos A. K. Singh I. Francis M. A. Moriarty H. Pike M. J. (2003). Dietary flavanols and procyanidin oligomers from cocoa (*Theobroma cacao*) inhibit platelet function. *Am. J. Clin. Nutr.* 77 1466–1473. 10.1093/ajcn/77.6.1466 12791625

[B35] Ncd Risk Factor Collaboration (Ncd-RisC) (2017). Worldwide trends in blood pressure from 1975 to 2015: a pooled analysis of 1479 population-based measurement studies with 19⋅1 million participants. *Lancet* 389 37–55. 27863813 10.1016/S0140-6736(16)31919-5 PMC5220163

[B36] Nerenberg K. A. Zarnke K. B. Leung A. A. Dasgupta K. Butalia S. McBrien K. (2018). Guidelines hypertension Canada’s 2018 guidelines for diagnosis, risk assessment, prevention, and treatment of hypertension in adults and children. *Can. J. Cardiol.* 34 506–525. 29731013 10.1016/j.cjca.2018.02.022

[B37] Njike V. Y. Faridi Z. Shuval K. Dutta S. Kay C. D. West S. G. (2011). Effects of sugar-sweetened and sugar-free cocoa on endothelial function in overweight adults. *Int. J. Cardiol.* 149 83–88. 10.1016/j.ijcard.2009.12.010 20036019

[B38] Organisation Mondiale de la Santé Genève (OMS) (2011). *Causes of Death 2008 [Base de Données en Ligne].* Available online at: https://www.who.int/healthinfo/global_burden_disease/cod_2008_sources_methods.pdf (accessed July 12, 2020).

[B39] Ozkor M. A. Rahman A. M. Murrow J. R. Kavtaradze N. Lin J. Manatunga A. (2014). Differences in vascular Nitric oxide and endothelium-derived hyperpolarizing factor bioavailability in African Americans and Whites. *Arterioscler. Thromb. Vasc. Biol.* 34 1320–1327. 10.1161/atvbaha.113.303136 24675657 PMC4138537

[B40] Ried K. Sullivan T. Fakler P. Frank O. R. Stocks N. P. (2010). Does chocolate reduce blood pressure? A meta-analysis. *BMC Med.* 8:39. 10.1186/1741-7015-8-3 20584271 PMC2908554

[B41] Ried K. Sullivan T. R. Fakler P. Frank O. R. Stocks N. P. (2017). Effect of cocoa on blood pressure. *Cochrane Database Syst. Rev.* 4:CD008893. 10.1002/14651858.CD008893.pub3 28439881 PMC6478304

[B42] Shiina Y. Funabashi N. Lee K. Murayama T. Nakamura K. Wakatsuki Y. (2009). Acute effect of oral flavonoid-rich dark chocolate intake on coronary circulation, as compared with non-flavonoid white chocolate, by transthoracic Doppler echocardiography in healthy adults. *Int. J. Cardiol.* 131 424–429. 10.1016/j.ijcard.2007.07.131 18045712

[B43] Siransy-Balayssac E. Ouattara S. Yeo T. A. Kondo A. Y. Toure M. Dah C. S. (2020). Physiological variations of blood pressure according to gender and age among healthy young black Africans aged between 18 and 30 years in Cote d’Ivoire, West Africa. *Physiol. Rep.* 8:e14579. 10.14814/phy2.14579 PMC7521662 32986938

[B44] Society of Actuaries (1959). *Build and Blood Pressure Study.* Chicago: Society of Actuaries.

[B45] Sudano I. Flammer A. J. Roas S. Enseleit F. Ruschitzka F. Corti R. (2012). Cocoa, blood pressure, and vascular function. *Curr. Hypertens. Rep.* 14 279–284. 10.1007/s11906-012-0281-8 22684995

[B46] Taubert D. Roesen R. Lehmann C. Jung N. Schomig E. (2007a). Effects of low habitual cocoa intake on blood pressure and bioactive nitric oxide: a randomized controlled trial. *JAMA* 298 49–60. 10.1001/jama.298.1.49 17609490

[B47] Taubert D. Roesen R. Schömig E. (2007b). Effect of cocoa and tea intake on blood pressure: a meta-analysis. *Arch. Intern. Med.* 167 626–634. 10.1001/archinte.167.7.626 17420419

[B48] Unger T. Borghi C. Charchar F. Khan N. A. Poulter N. R. (2020). 2020 International Society of Hypertension global hypertension practice guidelines. *J. Hypertens.* 38 982–1004. 10.1097/hjh.0000000000002453 32371787

[B49] Wood J. E. Senthilmohan S. T. Peskin A. V. (2002). Antioxydant activity of Procyanidin containing plant extracts at different PH. *Food Chem.* 77 155–161. 10.1016/s0308-8146(01)00329-6

[B50] Yaya H. S. Kengne A. P. (2014). *Le Défi de la Prévention Des Maladies Cardiovasculaires et ses Perspectives en Afrique. (The Challenge of Preventing Cardiovascular Diseases and its Prospects in Africa).* Québec City, QC: Presses de l’Université Laval, 1–16.

